# Pharmacokinetics and relative bioavailability of an oral amoxicillin-apramycin combination in pigs

**DOI:** 10.1371/journal.pone.0176149

**Published:** 2017-04-20

**Authors:** Chongshan Dai, Tingting Zhao, Xing Yang, Xilong Xiao, Tony Velkov, Shusheng Tang

**Affiliations:** 1 Department of Pharmacology and Toxicology, College of Veterinary Medicine, China Agricultural University, Beijing, P. R. China; 2 Department of Drug Delivery, Disposition and Dynamics, Monash Institute of Pharmaceutical Sciences, Monash University, Victoria, Australia; Southwest University, CHINA

## Abstract

A new compound granular premix of amoxicillin (20% w/w dry mass)/apramycin (5% w/w dry mass) was developed, and its pharmacokinetics and relative bioavailability were determined in pigs following oral administration following a cross-over study design. The pharmacokinetic parameters of amoxicillin (t_1/2λ_ = 6.43 ± 4.85h, C_max_ = 3.2 ± 1.35 μg·mL^-1^, T_max_ = 1.92 ± 0.58, AUC_INF_ = 8.98 ± 2.11 h·μg·mL^-1^) and apramycin (t_1/2λ_ = 8.67±4.4h, C_max_ = 0.23 ± 0.12 μg·mL^-1^, T_max_ = 2.25 ± 0.82 h, AUC_INF_ = 12.37 ± 8.64h·μg·mL^-1^) when administered as the amoxicillin-apramycin granular premix did not significantly differ from those for the single-ingredient powder form of each component. The relative bioavailability of amoxicillin following oral administration of the amoxicillin-apramycin granular premix was 22.62% when compared to the intramuscular administration of commercial amoxicillin sodium-powder. This is the first report of a new amoxicillin-apramycin combination which has a potential veterinary application the for prevention and treatment digestive tract infections in pigs.

## Introduction

Antibiotics are an effective means for preventing disease and improving feed efficiency in agricultural animals, such as pigs [1]. Amoxicillin (AMO), a semi-synthetic penicillin derivative, has been widely used in veterinary and human medicine due to its broad antibacterial spectrum against both Gram-negative and Gram-positive pathogens [2, 3]. Unfortunately, AMO monotherapy has proven to be less effective in recent times due to the wide-spread occurrence of multi-drug resistant (MDR) bacteria [4, 5]. Antibiotic combination therapy is a far more effective approach for combating MDR pathogens and preventing the emergence of resistance commonly seen with monotherapy [4, 6]. A very effective therapeutic approach is to combine antibiotics with different modes of action. For example, it has demonstrated that the combination AMO (a cell wall synthesis inhibitor) with antibiotics that act on different cellular targets such as gentamicin [4], colistin [6] and ceftriaxone [7], has been shown to have a marked synergistic effect. Intuitively, we took the novel approach to combine AMO with apramycin (APR), an aminoglycoside which displays a unique bicyclic sugar moiety and a 4-monosubstituted 2-deoxystreptamine ring [8]. Notably, the combination of AMO-APR has been demonstrated to display good synergistic activity against *Staphylococcus aureus*, *Salmonella spp*., and *Escherichia coli* in a mouse thigh infection model [9]. In this report we describe the preparation of an AMO-APR granular premix and the determination of the pharmacokinetics and relative bioavailability in pigs. Our findings highlight the strong potential of this novel premix for use in veterinary medicine as a feed additive for the prevention and treatment of digestive tract infections in pigs.

## Materials and methods

### Chemicals

Amoxicillin (purity≥86%) and Apramycin sulfate (560 IU per mg) standard were purchased from the China Institute of Veterinary Drug Control (Beijing, China). Amoxicillin trihydrate powder (purity ≥ 99.8%) for oral administration and Amoxicillin sodium-powder (Amo Jian^®^) for intramuscular use were from Hebei Yuanzheng Pharmaceutical Co., Ltd. (Shijiazhuang, China). Apramycin sulfate powder (556 IU·mg^-1^) was purchased from Wuhan Huifengda Biological Technology Co., Ltd. (Wuhan, China). Medicinal starch and microcrystalline cellulose (MCC, PH101) were purchased from Beijing Fengli Jingqiu Commerce and Trade Co., Ltd. (Beijing, China). All other reagents were obtained from the Beijing Chemical Reagents Company (Beijing, China).

### Animals

Twelve healthy Landrace X Large White hybrid pigs (half male and half female) at an average body weight of 18.6 ± 4.2 kg (range 14–23 kg), were randomly divided into two groups (3 male and 3 female in each group). Pigs were housed individually in 12 pens and observed once per week before the experiments. The animals were given access to antibiotic free commercial-feed and water *ad libitum*. All animal experiments were approved by the China Agricultural University Animal Care and Use Committee.

### Preparation of the AMO-APR granular premix

The AMO-APR granular premix was prepared using an extrusion–spheronisation method, according to the Animal Pharmacopoeia of the people's Republic of China [10]. The procedure involves several distinct preparation phases, as follows: a uniform powder mixture of AMO-APR and excipients [starch: microcrystalline cellulose = 2:8; (m:m)] were wet massed by the addition of a liquid binder [0.5% carboxy methyl cellulose sodium (m/v)], followed by pressing of the moistened mass through a dome-shaped extrusion screen to form cylindrical extrudates (extrusion), which were then broken into smaller cylindrical rods and rounded into spherical granules in a spheroniser equipped with a friction plate with cross-hatched geometry (spheronisation); and finally dried in a fluid-bed drier (Chongqing Enger Granulating & Coating Technology Co., Ltd.).

The optimized formulation was obtained by evaluating the total yield of target granules, the planar critical angle and angle of repose as the indices. After excipient ingredients were determined, an orthogonal design experiment arranged in a L_9_ (3^4^) orthogonal table was used to investigate three factors: (i) the amount of liquid binder, (ii) spheronization speed and (iii) spheronization time, in which each factor had three levels. The optimized formulation consisted of: the liquid binder: 1 mL per gram of premix, spheronization speed, 1500 rpm/min, spheronization time, 1 min.

### Quality control of the AMO-APR granular premix

The optimized processes were as follows: AMO trihydrate (20%, w/w dry mass), APR (5%, w/w dry mass), medicinal starch and microcrystalline cellulose were mixed (batch size 250 g) at a mixing speed of 16 rpm for 20 minutes in a V-type blender (Taizhou Jincheng Pharmaceutical Machinery Co., Ltd.). Then the powder mixture was unloaded into a large tray and liquid binder (0.5% sodium carboxymethyl cellulose, CMC-Na) was added slowly during constant kneading, and the mixture was kneaded for 5 min. To ensure uniform binder distribution during wet blending, the material adhering to the mixing tray was regularly removed. The kneaded mass was subjected to extrusion by a single screw extruder equipped with a dome-shaped extrusion screen (perforation diameter: 0.4 mm) at a constant extrusion speed of 70 rpm. The extrudates were spheronised for 1 min on a 350 mm diameter friction plate rotating at 1500 rpm. Afterwards, the wet granules were dried for 60 min at 40°C in a fluid bed drier and then sieved through a vibrating screen in order to achieve the specified particle size (300–600 μm). The final premix contained AMO (as the active ingredient) 200 g·kg^-1^ and APR sulfate 50 g·kg^-1^.

### Animal experiments

#### Pharmacokinetics

A three-way-crossover study design was employed, comparing the pharmacokinetics of AMO and APR as single components and in combination with the common carrier (starch). A one week wash-out period was observed between each crossover administration, necessitating nearly 4 weeks of involvement. Each pig was randomly subjected to the following treatments: (i) AMO-APR granular premix (GRANULE_amo + apr_), (ii) a common carrier (starch) powder premix containing only AMO (POWDER_amo_) and (iii) containing only APR (POWDER_apr_). All the three products were administered intra-gastrically dissolved in 20 mL of water in a lubricious tube at a dosage of AMO 16 mg·kg^-1^ body weight (b.w.) or APR 4 mg·kg^-1^ b.w. The animals were fasted for 17 h before premix administration, and were fed 2 h after administration. Heparinised blood samples were collected through precaval vein at 0.25, 0.5, 0.75, 1, 1.5, 2, 3, 3.5, 4, 5, 6, 8, 10, 12, 14 and 24 h after AMO and APR administration; blood samples were collected before administration as a blank control. Blood samples were centrifuged at 3000 *× g* for 15 min and the plasma samples were stored at -80°C until analysis.

#### Relative bioavailability

The relative bioavailability of AMO in the AMO-APR granular premix was investigated by a crossover study including orally (p.o.) administration of the AMO-APR granular premix and intramuscular (i.m.) administration AMO sodium. After one week wash-out period, pigs were randomly subjected to the following two treatments: (i) AMO-APR granular premix (GRANULE_amo + apr_) at a dose of 16 mg·kg^-1^ AMO. Blood samples were collected at 0.25, 0.5, 0.75, 1, 1.5, 2, 3, 3.5, 4, 5, 6, 8, 10, 12, 14 and 24 h after administration; and before administration as a blank control. (ii) Commercially available AMO sodium for injection (Amo Jian^®^) at a dose of 16 mg·kg^-1^. The product was freshly prepared in distilled water for intramuscularly administered in the neck. Blood samples were collected as described as 0.13, 0.25, 0.5, 0.75, 1, 1.5, 2, 3, 3.5, 4, 5, 6, 8, 10, 12, 14 and 24 h after administration.

### Analytical methods

#### Extraction of AMO from plasma and HPLC analysis

For sample extraction and clean-up, a 0.3 mL plasma sample was used, protein precipitation was achieved by the addition of 1.4 mL of 0.2% formic acid in acetonitrile solution (first with 1% formic acid in water, then adding 20% of 1% formic acid to acetonitrile (v/v), vortexed for 2 min and then centrifuged at 13680 × *g* for 25 min. The supernatant was transferred into a 2 mL centrifuge tube and evaporated to dryness under a gentle nitrogen stream at 40°C. The residue was reconstituted with 0.3 mL of water, vortexed for 2 min and centrifuged at 13680 × *g* for 15 min at 4°C. The supernatant was then aliquoted into injection vials and 50 μL was injected into the HPLC system, analyzed by using Waters 600-717-2487 system (Waters, Co., LTD) fitted with a reversed-phase column (SYMMETRY SHIELD RP 18, 4.6 × 250 mm i.d., 5 μm, Waters Co., USA). Detection was effected with a UV detector set at 230 nm. The mobile phase consisted of water, formic acid and acetonitrile (1000:2:20, v/v/v) at a flow rate of 1.0 mL·min^-1^ at room temperature. Standard curves of peak area versus AMO concentration were linear from 0.075–10 μg·mL^-1^ by analysis of six spiked plasma samples and the correlation coefficients (r) were >0.999 for calibration curves. The intra-day RSD% values for AMO were in the range of 3.91–6.08% while the values for inter-day were in the range of 3.82%-7.44% at three quality control samples of 0.075, 1.0 and 10 μg·mL^-1^ on three consecutive days. The limits of detection (LOD) and quantification (LOQ) for AMO were 0.025 and 0.075 μg·mL^-1^, respectively.

#### Extraction of APR from plasma and HPLC analysis

Plasma samples (0.5 mL) were treated with 1.0 mL 10% TCA (containing 0.04 mM Na_2_EDTA, w/v). The sample was vortex mixed for 2 min then centrifuged at 9500 *× g* for 10 min at 4°C. The clear supernatant was transferred into a 5 mL centrifuge tube and the precipitate was extracted once again using the above method, incorporating the twice extract supernatant for the PE procedure. A mixed solid-phase extraction cartridge (Oasis MCX 30 mg, 1 cc, Waters) was conditioned with 1 mL methanol followed by 2 mL water. All of the extract solution was applied to the column, which was then washed with 2 mL water followed by 1 mL 0.5% ammoniated methanol solution (v/v). The cartridge was dried under vacuum then eluted into a 10 mL centrifuge tube with 3 mL 5% (v/v) ammoniated methanol solution and dried. The eluate was collected was evaporated to dryness at 50°C water bath under a gentle stream of nitrogen gas, then 0.5 mL borate buffer solution (0.012 M, pH 9.0) was added and vortex mixed for 1 min. Then 0.5 mL FMOC-Cl (2.0 mM in aceonitrile) was added to the tube and vortexed for 30 sec, the mixture was left to react at ambient temperature in the dark for 20 min, then 50 μL glycine (0.1 M) was added to stop the reaction. The final mixture was transferred into 1.5 mL micro-centrifuge tube and centrifuged at 9500 *× g* for 5 min at 4°C, the supernatant was aliquoted into an auto-sampler vial and 20 μL was injected for HPLC analysis. HPLC was performed using a Waters 2695 HPLC (Waters, Co., LTD) modular system fitted with a reversed-phase column (SYMMETRY SHIELD RP 18, 4.6 × 250 mm i.d., 5 μm, Waters Co., LTD, USA). A 2475 fluorescence detector was used for detection at an excitation wavelength of 260 nm and an emission wavelength of 315 nm. Separation was performed using acetonitrile-water (77:23, v/v) as the mobile phase, at a flow rate of 1.0 mL/min and the column oven temperature was set at 30 ± 5°C. Calibration curves for APR were constructed from a serial of blank plasma spiked with six different concentrations and the correlation coefficients (r) were >0.999 from 0.05–2 μg/mL. The intra-day precision for APR was 3.40–6.68% and the value for inter-day was 4.92–8.17% at three quality control samples of 0.05, 0.5 and 2 μg·mL^-1^ on three consecutive days. The limit of detection (LOD) for APR was 0.015 μg·mL^-1^ and the limit of quantification (LOQ) was 0.05 μg·mL^-1^.

### Statistical analysis

Pharmacokinetic analysis of the data was performed using non-compartmental analysis by the software data package Phoenix WinNonlin 6.1 (Pharsight Corporation, Mountain View, CA, USA). The parameters determined include λz (the slope of the terminal elimination phase of the time-concentration curve), t_1/2λ_ (elimination or terminal half-life), C_max_ (maximum plasma concentration), T_max_ (time to reach C_max_), AUC_INF_ (area under the plasma concentration–time curve from time zero to infinity), AUMC_INF_ (area under the moment curve), MRT (mean residence time). All of these parameters were compared between the different products by one way analysis of variance (ANOVA) using statistical software SPSS 17.0 (SPSS Inc., Chicago, IL, USA). All results were presented as mean ± SD and a *P*-value of 0.05 was set as the threshold for differences to be considered statistically significant. The relative bioavailability (F) of AMO was calculated by the method of corresponding areas, which entails comparison of the total areas under the plasma concentration-time curves (AUC) obtained after p.o and i.m. administration: F = AUC_p.o._/AUC_i.m._.

## Results

### Preparation of AMO-APR granular premix

After testing and optimising the preparation process and appropriate ratio of the excipients, a novel AMO-APR granular premix was successfully developed. The roundness (14.0–14.8 φ/°) and liquidity (19.9–20.7 α/°) of the granules all satisfied the Technical Standards of Ministry of Agriculture of People’s Republic of China.

### Comparative pharmacokinetics of AMO alone and in the AMO-APR granular premix

**Fig 1:**
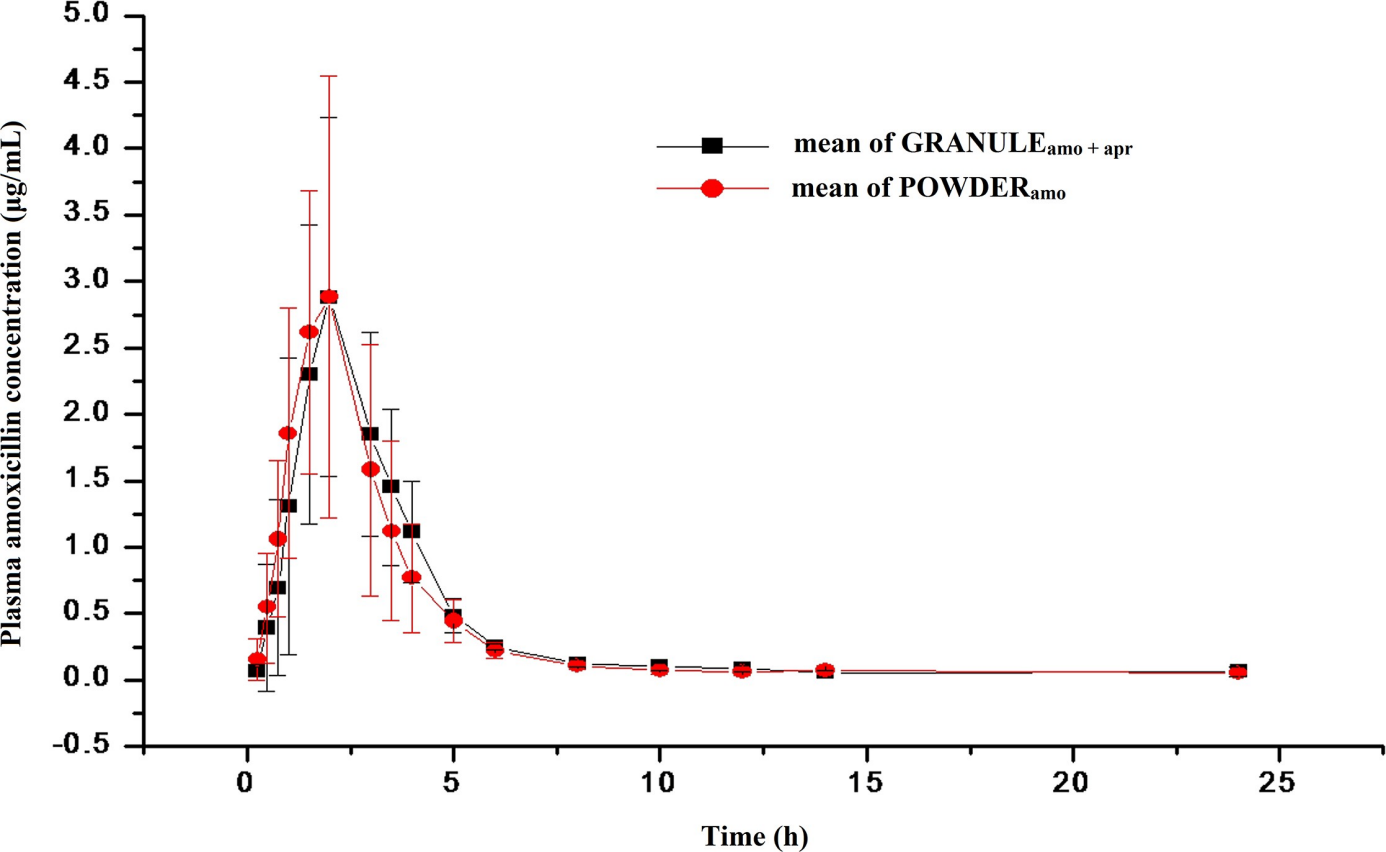
Plasma concentration of amoxicillin (μg·mL^-1^) in pigs after oral administration at a dosage of 16 mg·kg^-1^ amoxicillin. Values are presented as mean ± SD (n = 6).

The time to the mean concentration of AMO in plasma after oral administration as AMO powder (POWDER_amo_) and as the AMO-MPR granular premix (GRANULE_amo + apr_) at a dosage of 16 mg·kg^-1^ are depicted in Fig 1. Essentially, the curves for both formulations are characterized by a rapid, albeit, only partial absorption of AMO and a biphasic decline in the concentration over time. The corresponding pharmacokinetic parameters derived from a noncompartmental analysis of the data are presented in Table 1. There was no significant difference (*P* > 0.05) in the pharmacokinetic characteristics between the GRANULE_amo + apr_ and POWDER_amo_. The mean peak plasma concentration was achieved at ~1.92 h with a C_max_ of ~3.20 μg·mL^-1^ for both formulations and the AUC_INF_ was 8.98 and 8.43 h·μg·mL^-1^ for the GRANULE_amo + apr_ and POWDER_amo_ formulations, respectively.

**Table 1:** Pharmacokinetic parameters of amoxicillin in pigs (n = 6) after oral administration at dose of 16 mg·kg^-1^.

Parameters	AMO in GRANULE_amo + apr_		AMO in POWDERamo		
	Mean	SD	Mean	SD	*P*-value
λz (1h^-1^)	0.17	0.11	0.18	0.12	0.82
t_1/2λ_ (h)	6.43	4.85	6.33	4.93	0.97
C_max_ (μg·mL^-1^)	3.2	1.35	3.25	1.47	0.95
T_max_(h)	1.92	0.58	1.92	0.58	1
AUC_INF_(h·μg·mL^-1^)	8.98	2.11	8.43	3.04	0.72
AUMC_INF_(h^2^·μg·mL^-1^)	36.18	13.08	27.99	8.22	0.22
MRT(h)	3.97	0.79	3.43	0.87	0.29

### Comparative pharmacokinetics of of APR alone and in the AMO-APR granular premix

**Fig 2:**
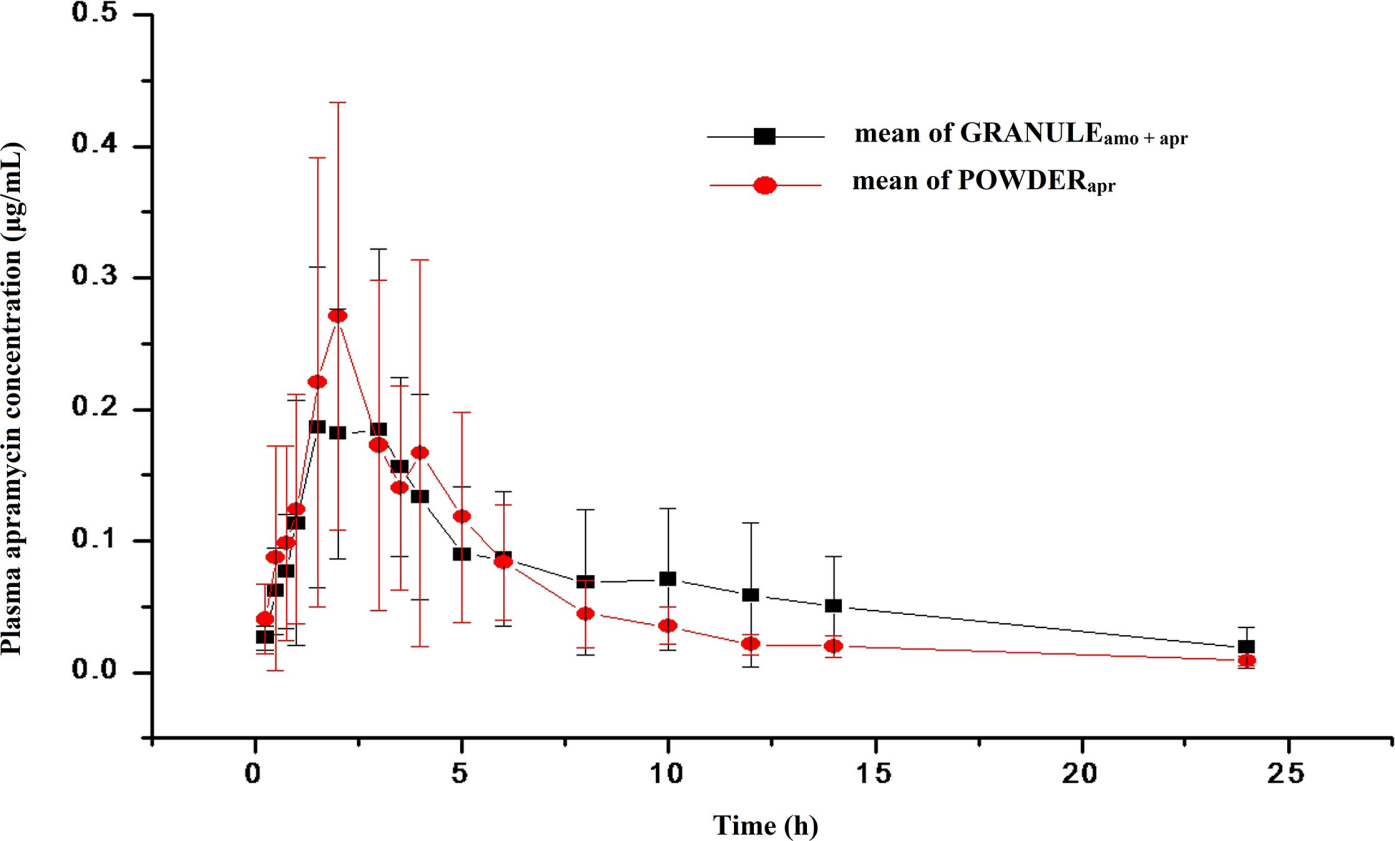
Plasma concentration of apramycin (μg·mL^-1^) in 6 pigs after oral administration at a dosage of 4 mg·kg^-1^ apramycin. Values are presented as mean ± SD (n = 6).

The time to mean concentration of APR in plasma after oral administration as APR powder (POWDER_apr_) and as the AMO-MPR granular premix (GRANULE_amo + apr_) at a dosage of 4 mg·kg^-1^ are depicted in Fig 2. The corresponding pharmacokinetic parameters on the basis of noncompartmental analysis are documented in Table 2. The curves for both formulations indicate APR is poorly absorbed from the gastrointestinal tract and there were no significant differences (*P* > 0.05) in pharmacokinetics between GRANULE_amo + apr_ and POWDER_apr._ (Table 2). The mean C_max_ of APR from GRANULE_amo + apr_ was 0.23 μg·mL^-1^ and was reached at 2.25 h, while the mean C_max_ of the POWDER_apr_ was 0.29 μg·mL^-1^ and was reached at 2.33 h. The AUC_INF_ for GRANULE_amo + apr_ and POWDER_apr_ were 1.59 and 1.31 h·μg·mL^-1^, respectively (Table 2).

**Table 2:** Mean and standard deviation of pharmacokinetic parameters of apramycin in pigs (n = 6) after oral administration at dose of 4 mg·kg^-1^.

Parameters	APR in GRANULE_amo + apr_		APR in POWDER_amo_		
	Mean	SD	Mean	SD	*P*-value
λz (1h^-1^)	0.1	0.06	0.1	0.04	0.98
t_1/2λ_ (h)	8.67	4.4	7.81	3.43	0.71
C_max_ (μg·mL^-1^)	0.23	0.12	0.29	0.18	0.53
T_max_(h)	2.25	0.82	2.33	1.54	0.91
AUC_INF_(h·μg·mL^-1^)	1.59	1	1.31	0.64	0.58
AUMC_INF_(h^2^·μg·mL^-1^)	12.37	8.64	7.14	3.3	0.2
MRT(h)	7.66	1.64	5.73	1.38	0.06

### Relative bioavailability of AMO in the compound AMO-APR granular premix

**Fig 3:**
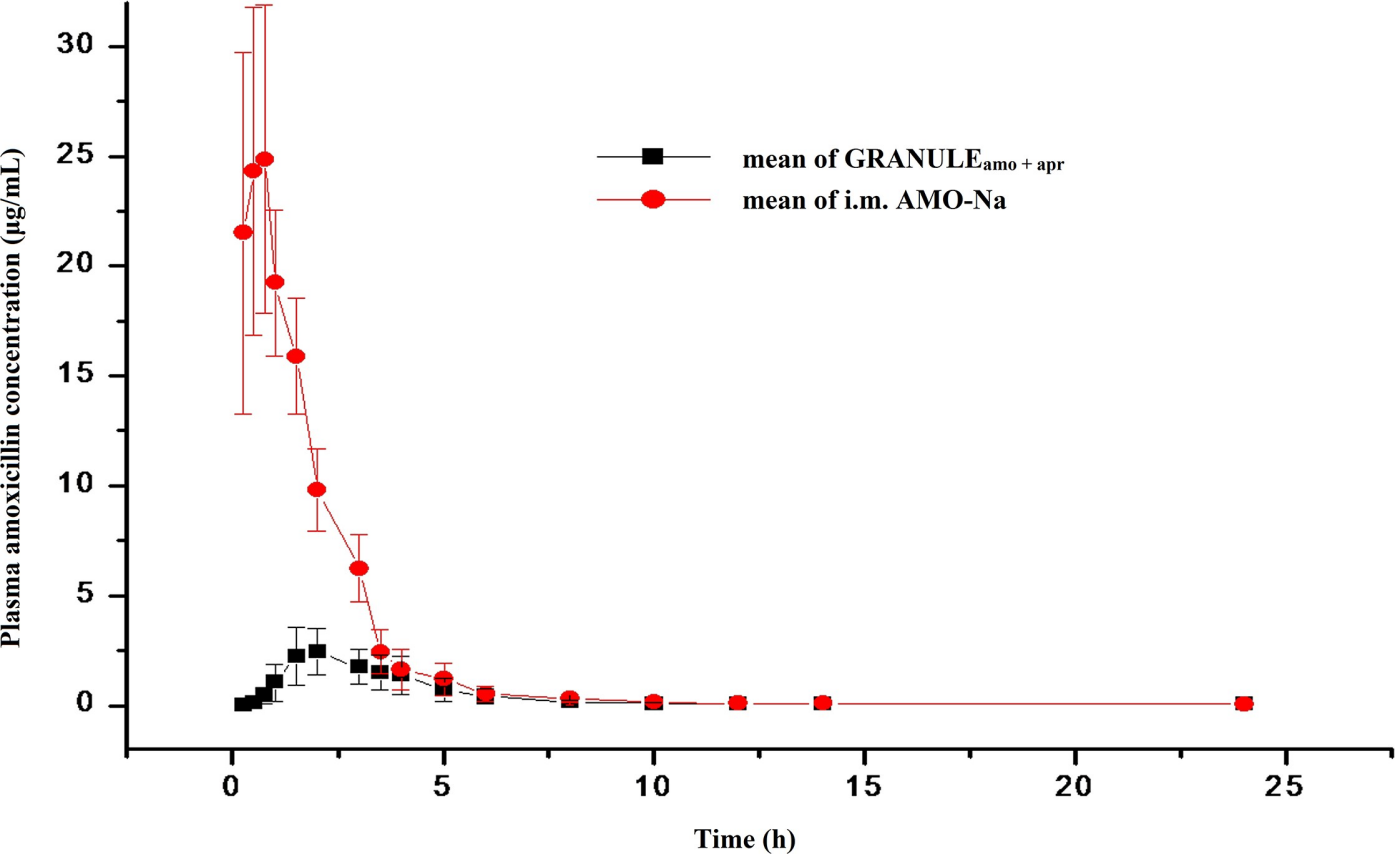
Plasma concentration of amoxicillin (μg·mL^-1^) in 6 pigs after oral and intramuscular administration at a dosage of 16 mg·kg^-1^ amoxicillin. Values are presented as mean ± SD (n = 6).

The times to mean concentration for AMO following p.o administration as the GRANULE_amo + apr_ *versus* intramuscular administration (I.M._amo_) at a dose of 16 mg·kg^-1^ are presented in Fig 3. Following intramuscular administration, a rapid absorption of AMO from the injection site occurs, reaching a C_max_ of 27.20 μg·mL^-1^ at 0.44 h. The pharmacokinetic parameters after p.o. and i.m. administration are documented in Table 3. The relative bioavailability of AMO (AUC_p.o._/AUC_i.m._) was 22.62%.

**Table 3:** Pharmacokinetic parameters of amoxicillin in pigs (n = 6) after intramuscular and oral administration at dose of 16 mg·kg^-1^.

Parameters	AMO in GRANULE_amo + apr_		AMO in POWDER_amo_		
	Mean	SD	Mean	SD	*P*-value
λz (1h^-1^)	0.16	0.14	0.2	0.11	0.66
t_1/2λ_ (h)	7.3	5	4.17	1.5	0.17
C_max_ (μg·mL^-1^)	2.85	0.74	27.2	7.36	<0.001
T_max_(h)	2.17	1.08	0.44	0.31	0.004
AUC_INF_(h·μg·mL^-1^)	8.95	2.8	39.16	3.13	<0.001
AUMC_INF_(h^2^·μg·mL^-1^)	35.58	19.02	57.23	13.8	0.048
MRT(h)	3.83	0.99	1.46	0.33	<0.001
F(%)	22.62	5.48			

## Discussion

Piglet’s diarrhea due to mixed bacterial infections is a prevalent problem worldwide [11]. The use of antibiotic combinations is of considerable utility for the treatment of mixed infections and the prevention of resistance [12]. AMO has been shown to display good antibacterial activity against pathogens such as *E*. *coli* and *Salmonella spp*. in digestive tract infections [13]. Similarly, APR has been validated for the treatment of intestinal bacterial infections in pigs and chickens [14–16]. In our *in vitro* pilot study, the combination of AMO with APR produced an additive effect (FIC index > 0.5 to 1) against *Staphylococcus aureus* (ATCC25923), *Salmonella spp*. (CMCC50115) and *E*. *coli* (ATCC35218) (unpublished data). In the present study, we describe the development of a novel AMO-APR formulation with the potential veterinary application as an in-feed premix to prevent and treat digestive tract infections in pigs.

Currently available commercial single-ingredient AMO and APR products are mostly in powdered form. The commercially available single component feed powder for AMO recommends a 15–20 mg·kg^-1^ b.w dosage to pigs via p.o. administration; and 4–8 mg·kg^-1^ b.w. for APR (Apralan G + 200 Premix) [17]. In the present study, we formulated the dosage of GRANULE_amo + apr_ as 16 mg and 4 mg per kg b.w., which is consistent with the recommended dosage of Commission of Chinese Veterinary Pharmacopoeia [10]. A previous study demonstrated a significant reduction of bioavailability and 18.5% increase in renal clearance after oral administration of AMO combined with sodium diclofenac [18]. In contrast, our study showed that the AMO-APR granular premix possesses the same pharmacokinetic characteristics (t_1/2λ_, C_max_, T_max_, AUC_INF_, MRT) when compared to the single-ingredient powdered form of each antibiotic (Tables 1 and 2). Overall, our results indicated that there was no interaction between AMO and APR when they were administered orally to pigs as the granular premix.

For AMO, the pharmacokinetic parameters after oral administration of GRANULE_amo + apr_ reached a peak plasma concentration of 3.20 μg·mL^-1^, ~1.92 h post-administration and the AUC_INF_ was 8.98 h·μgmL^-1^, which is markedly different compared to previously published data for oral AMO in pigs from Hernandez *et al*. (C_max_ = 0.76 μg·mL^-1^ and t_max_ = 5.78 h) and Martinez-Larranaga *et al*. (C_max_ = 7.37 μg·mL^-1^ and t_max_ = 0.97 h) [19, 20].The discrepancy maybe related to differences in the varied methods of oral administration of AMO and its different formulation (i.e powder, granulation) [21]. In the present study, the peak plasma concentration of APR was 0.23 μg·mL^-1^ and reached at 2.25 h, the AUC_INF_ was 1.59 h·μg·mL^-1^ when pigs were orally administrated with GRANULE_amo + apr_ at 4 mg·kg^-1^. The lower C_max_ and AUC_INF_ were consistent with a previous study in pigs [22] and in broiler chickens [23]; This suggests that APR is poorly absorbed across the gastrointestinal tract. In addition, the slight slow release of APR [MRT extensions from 5.73 to 7.66 (*P* = 0.06)] in formation of GRANULE_amo + apr_ was observed, which may be relevant with the formation changes of APR. Nevertheless, the formulation is still of utility for gastrointestinal infections [24].

Previous studies have showed the oral bioavailability of AMO can be variable, which may be associated with the different methods of oral administration[19] [25]. In the present study, the relative bioavailability of AMO in fasted-pigs following gavage administration of GRANULE_amo + apr_ at 16 mg·kg^-1^ compared to i.m. administration was 22.62% based on the values of AUC_INF_ (8.98 h·μg·mL^-1^: 39.16 h·μg·mL^-1^) (Table 3). Agersø and Friis’ showed that the bioavailability of AMO following intramuscular administration (14.1 mg·kg^-1^) in pigs is 83%; whereas the relative bioavailability of AMO in pigs following p.o. administration (10 mg·kg^-1^) (AUC_INF_ = 6.5 h·μg·mL^-1^) compared to i.m. administration was 27.39%[25]. Hernandez *et al*. reported that the bioavailability of AMO (AMO 10% Premix) in pigs following administration in-feed medication at 15 mg·kg^-1^ was 11% [19]. Similarly, Anfossi *et al*. [26] reported an AUC_INF_ of 12.1 h·μg mL^-1^ for AMO (50 mg·kg^-1^ b.w.) orally administered to pigs by in-feed medication, which is only two-fold higher than the AUC_INF_ values reported by Agersø and Friis [25] and 1.35-fold higher than that in the present study. This variability may be attributable to the absorption of AMO across the intestinal mucosa by both passive diffusion and active transport processes [19, 25–27]. It also contributed to explain the non-sigificantance (*P* values among 0.22 to 1; Table 1) of all pharmacokinetic parameters between oral administration of GRANULE_amo + apr_ and POWDER_amo_ in pigs, although the minor changes was present with the different formulation of AMO.

Based on previously reported values of AUC_INF_ (4.1 h·μg·mL^-1^: 130.6 h·μg·mL^-1^) after p.o. administration (20 mg·kg^-1^ b.w.) [22] and our results (AUC_INF_ = 1.59 h·μg·mL^-1^ when p.o. administration at 4 mg·kg^-1^ b.w), it appears APR displays a much lower bioavailability (~3%) compared to AMO. Overall, the incomplete and poor absorption of AMO and APR following oral administration of the premix indicates most of the drugs remain in the gastrointestinal tract, which is beneficial for the treatment of gastrointestinal infections. Notably, AMO has been widely used to treat respiratory infections in pigs caused by *Actinobacillus pleuropneumoniae* and *Streptococcus suis* (MIC_90_ was <0.25 μg·mL^-1^) [28]. One of the dosing criteria is that the plasma concentration should exceed the MIC for the whole or at least half of the inter-dose interval [29]. The present study showed that a mean plasma concentration above 0.25 μg·mL^-1^ was maintained ~6 h following p.o. administration of GRANULE_amo + apr_, indicating that oral administration of the new formulation at 16 mg·kg^-1^, twice daily may be applicable for the treatment of respiratory tract infections in pigs.

In conclusion, this is first report of the formulation and pharmacokinetics in pigs of a novel AMO-APR in-feed premix that may be of use to control intestinal and respiratory tract infections in pigs. The next step would be to perform large-scale studies to determine the efficacy and develop the first scientifically-based dosing recommendations.

## Supporting information

S1 Checklist NC3Rs ARRIVE guidelines checklist. (PDF)
